# Measuring improvement in fracture risk prediction for a new risk factor: a simulation

**DOI:** 10.1186/s13104-018-3178-z

**Published:** 2018-01-22

**Authors:** Lisa M. Lix, William D. Leslie, Sumit R. Majumdar

**Affiliations:** 1 Department of Community Health Sciences, Rady Faculty of Health Sciences, University of Manitoba, Winnipeg, MB Canada; 2 Department of Internal Medicine, Rady Faculty of Health Sciences, University of Manitoba, Winnipeg, MB Canada; 3 Department of Medicine, Faculty of Medicine and Dentistry, University of Alberta, Edmonton, AB Canada

**Keywords:** Prediction, Statistical model, Fracture risk, Simulation

## Abstract

**Objective:**

Improvements in clinical risk prediction models for osteoporosis-related fracture can be evaluated using area under the receiver operating characteristic (AUROC) curve and calibration, as well as reclassification statistics such as the net reclassification improvement (NRI) and integrated discrimination improvement (IDI) statistics. Our objective was to compare the performance of these measures for assessing improvements to an existing fracture risk prediction model. We simulated the effect of a new, randomly-generated risk factor on prediction of major osteoporotic fracture (MOF) for the internationally-validated FRAX^®^ model in a cohort from the Manitoba Bone Mineral Density (BMD) Registry.

**Results:**

The study cohort was comprised of 31,999 women 50+ years of age; 9.9% sustained at least one MOF in a mean follow-up of 8.4 years. The original prediction model had good discriminative performance, with AUROC = 0.706 and calibration (ratio of observed to predicted risk) of 0.990. The addition of the simulated risk factor resulted in improvements in NRI and IDI for most investigated conditions, while AUROC decreased and changes in calibration were negative. Reclassification measures may give different information than discrimination and calibration about the performance of new clinical risk factors.

## Introduction

Methods to predict the risk of an outcome are receiving considerable attention in the clinical literature. The incremental improvement in risk prediction when a new risk factor is added to an existing model is of particular interest because new measures of risk are continually being defined and collected in an attempt to refine prediction models [1]. This is an important topic for osteoporosis-related fracture risk prediction, where a number of models have been proposed [2] and numerous studies have examined the incremental improvement in prediction when biomarkers or other clinical characteristics of patients are introduced to existing models [3, 4].

Improvements in predictive model performance have traditionally been assessed using area under the receiver operating characteristic (AUROC) curve, which measures model discrimination, and calibration, which measures prediction error. Using these measures, a new risk factor is considered to be a beneficial model addition if the AUROC and calibration statistics for the new model, which includes the predictors in the original model plus the new risk factor, are better than the corresponding statistics for the original model. However, AUROC and calibration statistics are summary measures that may not provide a complete picture of the change in predicted risk for all individuals [5], particularly those in the lowest and highest risk categories.

Clinicians have given increased attention to reclassification tables and statistics such as the net reclassification index (NRI), which summarize the change in risk probability or the frequency (percentage) of individuals who will move from one risk category to another based on the addition of a new risk factor to the original prediction model. Reclassification statistics are increasingly used to describe the performance of risk prediction models [4, 6, 7]. However, few studies, particularly in the area of fracture risk prediction, have compared the performance of different measures [8].

Our purpose was to compare conventional AUROC and calibration statistics with newer reclassification statistics for fracture risk prediction. We did this within the context of the internationally-validated Fracture Risk Assessment Tool (FRAX^®^), which predicts risk of a major osteoporotic fracture (MOF) [9]. Our hypothesis was that conventional measures and newer reclassification statistics would not lead to the same conclusions about the incremental improvement in model performance when a new risk factor was added to the FRAX^®^ model.

## Main text

### Methods

#### Study design and cohort development

The study was conducted by combining analyses of a real dataset with simulation. The real data were from the province of Manitoba, Canada for the period from 1987 to 2011 and came from the Manitoba Bone Mineral Density (BMD) Program and administrative health databases, including hospital separation records, physician billing claims, prescription drug records, and population registry.

The Manitoba BMD Program database is a regionally-based clinical database that captures dual energy X-ray absorptiometry (DXA) results for the entire provincial population since the program’s inception in 1996 [10]. Hospital abstracts are completed at the point of discharge from acute care facilities and contain diagnoses coded using the World Health Organization’s International Classification of Diseases (ICD). Physician claims are submitted to the provincial ministry of health by physicians paid on a fee-for-service basis; they capture virtually all outpatient services and contain a single ICD code. Prescription drug records are from the Drug Program Information Network, a centralized, electronic, point-of-sale database connecting all retail pharmacies. The population registry captures information on all provincial residents eligible to receive publicly-insured health services, including dates of health insurance coverage and demographics.

The study cohort included women aged 50+ years who had a BMD test between 1996 and 2011. If an individual had more than one BMD test during this period, only the first one was used. The BMD test date was the index date for creating predictors for the FRAX^®^ model: age, body mass index, prior fracture, parental hip fracture, chronic obstructive pulmonary disease, rheumatoid arthritis, alcohol or substance use, recent glucocorticoid use, and femoral neck T-score. These measures were defined from the Manitoba BMD Program database and codes in administrative health databases [11–15].

MOF encompasses fractures of the spine, hip, forearm, and humerus. Fractures that occurred after the index BMD test and up to March 31, 2011, death, or migration out of province, were identified from hospital and physician billing claims databases. Health service records were assessed for fracture information not associated with trauma using established methods [12].

The study cohort was described on socio-demographic and clinical characteristics using means, standard deviations, and percentages. The 10-year MOF risk was estimated for each cohort member using the FRAX^®^ Canada calculator (FRAX^®^ Desktop Multi-Patient Entry, version 3.7) [16]. FRAX^®^ uses a continuous hazard function based on Poisson regression to produce risk estimates.

#### Computer simulation

In the computer simulation, new risk predictions were generated based on the addition of one multiplicative risk factor to the original FRAX^®^ estimates. This risk factor was simulated from a Bernoulli distribution and was independent of other predictors; Kooter et al. [17] demonstrate formulae to estimate the impact of simulated risk factors on predicted risk. Relative risk (RR), which quantifies the independent association between this simulated variable and the outcome, varied from 1.25 to 3.50 in increments of 0.25. Prevalence varied from 10 to 100% in increments of 10%. The intervention threshold, which was used to construct the reclassification tables, ranged from 5 to 50% in increments of 5%.

#### Statistical analysis

For each combination of simulation parameters, AUROC and calibration statistics were computed for the original FRAX^®^ model and the new model. AUROC was calculated based upon the original and simulated risk predictions; the difference was computed. Calibration was the ratio of the observed cumulative fracture incidence at 10 years to the average predicted risk probability [1].

The NRI measures the frequency of appropriate reclassification compared to inappropriate reclassification with the new model compared to the original model. The predicted probabilities based on the two models are assigned to ordinal risk categories and cross-tabulated [5]. We defined upward movement as a change into a higher risk category based on the new model and downward movement as a change in the opposite direction. As per convention, for individuals with a fracture event, a value of 1 was assigned for upward movement, a score of − 1 was assigned for downward movement, and zero was assigned for no change. The opposite scoring was used for cohort members who do not experience a fracture event. The NRI is the sum of individual scores, divided by the number of cohort members. The IDI is based on the change in calculated risk; specifically, it quantifies the increment in the predicted probabilities for the cohort members experiencing an event and the decrement for the cohort members who do not experience an event [5, 18]. Conventional and reclassification statistics for the original and new models were descriptively analysed. Statistical analyses were conducted using SPSS for Windows, Version 22.0.

### Results

#### Study cohort characteristics

The study cohort (Table 1) was comprised of 31,999 women 50+ years of age. A total of 9.9% sustained a MOF; 17.2% were censored at death. There were differences between cohort members with and without a MOF on most variables in the original FRAX^®^ model. The 10-year estimated MOF risk from the original model was 10.5% [standard deviation (SD) = 6.8%] for cohort members without a MOF and 16.3% (SD = 9.6%) for cohort members with a MOF.

**Table 1:** Characteristics of the study cohort, overall and by fracture outcome

	Overall (*N* = 31,999)	No MOF (*N* = 28,817)	MOF (*N* = 3182)
Age (years)	65.6 ± 9.7	65.1 ± 9.6	70.2 ± 9.7
Body mass index (kg/m^2^)	26.7 ± 5.2	26.8 ± 5.2	25.7 ± 4.9
Prior fracture, n (%)	3977 (12.4)	3228 (11.2)	749 (23.5)
Parental hip fracture, n (%)	520 (1.6)	492 (1.7)	28 (0.9)
COPD, n (%)	3084 (9.6)	2648 (9.2)	436 (13.7)
Recent glucocorticoid use, n (%)	1502 (4.7)	1267 (4.4)	235 (7.4)
Rheumatoid arthritis, n (%)	1277 (4.0)	1065 (3.7)	212 (6.7)
Alcohol or substance abuse, n (%)	614 (1.9)	517 (1.8)	97 (3.0)
Femoral neck T-score	− 1.5 ± 1.0	− 1.4 ± 1.0	− 2.1 ± 0.9
FRAX^®^ MOF risk	11.1 ± 7.4	10.5 ± 6.8	16.3 ± 9.6
Follow up (years)	8.4 ± 2.8	8.4 ± 2.8	8.9 ± 2.8

All reported statistics are mean ± standard deviation unless otherwise noted; COPD, chronic obstructive pulmonary disease; MOF, major osteoporotic fracture; BMD, bone mineral density; all differences between sub-groups were statistically significant (*p* < 0.0001)

#### Risk prediction model characteristics and computer simulation

The estimated AUROC of the original model was 0.706 [95% confidence interval (95% CI) 0.697–0.716] and calibration was 0.990. Using this model, 6.8% of cohort members were predicted to have low fracture risk (i.e., < 10%), 13.9% as moderate fracture risk (i.e., 10–20%), and 27.3% as high fracture risk (i.e., > 20%).

The results obtained after introducing the new simulated risk factor into the original model are reported in Table 2. The first set of results was obtained when the RR varied and other parameters were held constant. Across the investigated RR values, the NRI demonstrated a U-shaped pattern; it was low for small values of the RR, increased for moderate values of RR, and then decreased for higher RR values. In fact, for RR > 3.0, the NRI attained a small negative value. In contrast, the IDI demonstrated small incremental increases as RR increased. The change in AUROC between the original and new model was negative; it decreased to a low of 0.642; in general, values less than 0.70 indicate poor discriminant performance [19]. Calibration also decreased as RR increased.

**Table 2:** Reclassification and conventional statistics for measuring change in FRAX^®^ model performance with the addition of a new simulated risk factor

Statistic	RR^a^									
	1.25	1.5	1.75	2.0	2.25	2.5	2.75	3.0	3.25	3.5
NRI	0.018	0.028	0.037	0.042	0.034	0.026	0.017	0.007	− 0.003	− 0.015
IDI	0.005	0.009	0.014	0.019	0.023	0.028	0.033	0.037	0.042	0.046
ΔAUROC	− 0.004	− 0.011	− 0.019	− 0.027	− 0.035	− 0.042	− 0.049	− 0.055	− 0.060	− 0.064
ΔCalibration	− 0.076	− 0.141	− 0.198	− 0.248	− 0.291	− 0.330	− 0.365	− 0.396	− 0.424	− 0.450

Statistic	Prevalence (%)^b^									
	10	20	30	40	50	60	70	80	90	100
NRI	0.015	0.025	0.037	0.048	0.063	0.073	0.085	0.098	0.107	0.120
IDI	0.006	0.010	0.017	0.022	0.029	0.036	0.042	0.048	0.053	0.058
ΔAUROC	− 0.010	− 0.020	− 0.025	− 0.029	− 0.029	− 0.026	− 0.021	− 0.015	− 0.009	0.000
ΔCalibration	− 0.090	− 0.165	− 0.228	− 0.283	− 0.330	− 0.371	− 0.408	− 0.440	− 0.469	− 0.495

Statistic	Intervention threshold (%)^c^									
	5	10	15	20	25	30	35	40	45	50
NRI	− 0.041	− 0.074	− 0.004	0.042	0.050	0.060	0.063	0.057	0.050	0.042
IDI	0.019	0.019	0.019	0.019	0.019	0.019	0.019	0.019	0.019	0.019
ΔAUROC	− 0.291	− 0.291	− 0.291	− 0.291	− 0.291	− 0.291	− 0.291	− 0.291	− 0.291	− 0.291
ΔCalibration	− 0.248	− 0.248	− 0.248	− 0.248	− 0.248	− 0.248	− 0.248	− 0.248	− 0.248	− 0.248

RR, relative risk; NRI, net reclassification index; IDI, integrated discrimination improvement

^a^The following simulation parameters were held constant: prevalence = 33% and intervention threshold = 20%

^b^The following simulation parameters were held constant: RR = 2.0 and intervention threshold = 20%

^c^The following simulation parameters were held constant: RR = 2.0 and prevalence = 33%

The second set of results was obtained when the prevalence of the new simulated risk factor was varied and other simulation parameters were held constant. The NRI increased from 0.015 to 0.120 as prevalence increased from 10 to 100%, while the IDI showed a more modest increase, from 0.006 to 0.058. The change in the AUROC was negative for all except the largest prevalence values and the change in calibration was negative for all conditions.

The final set of results, which was obtained by varying the intervention threshold for treatment, resulted in NRI values that ranged from − 0.041 to 0.063. Given that the IDI is based on continuous values of the risk probabilities, it did not change with variations in the intervention threshold, nor did the AUROC and calibration statistics.

### Discussion and conclusions

Several multivariable Fracture Risk Assessment Tools have been proposed [2], and there is continual exploration of new clinical risk factors that may improve fracture risk prediction in these tools [3]. There are multiple measures of improvement in predictive performance for a new risk factor. These measures will not always produce consistent results, confirming our hypothesis.

Our results show that a risk factor with a moderate to strong independent association with the outcome simultaneously resulted in decreases in model discrimination and calibration (demonstrating that a new risk factor does not always incrementally improve risk prediction) and positive changes in the NRI and IDI indicating improvements in risk classification. However, the NRI and IDI did not always produce consistent results. For example, as the NRI decreased the IDI increased when the RR of the new risk factor increased. However, when prevalence of the new risk factor increased, both the NRI and IDI increased. These findings are consistent with previous simulations [20].

This study, along with previous research about fracture risk prediction [21], underscores the importance of examining multiple performance measures in the development and refinement of fracture risk prediction models [7, 22]. Reclassification tables and statistics such as the NRI and IDI provide clinicians and researchers with supplementary statistical indicators about the potential uncertainty in risk estimates and the net effect of a new risk factor on predictive performance. The NRI and IDI can provide insights about scenarios for which appropriate reclassification occurs relative to inappropriate reclassification with the introduction of a new risk factor.

The benefits of adding a new risk factor to a prediction model such as FRAX^®^ will depend on a number of considerations, including cost, availability, and clinical relevance. Clinicians working in the area of fracture risk prediction, as in other risk prediction areas, must keep abreast of developments in risk modeling and continually look to new opportunities to add to their toolbox of relevant statistical methods.

## Limitations

The limitations of this study relate to the simulation and choice of statistical procedures. We manipulated a single risk factor in the simulation even though multiple risk factors might have been manipulated. However, researchers interested in improving risk prediction often focus on new risk factors one at a time [17]. We considered a dichotomous risk factor; ordinal or continuously-distributed risk factors could also be investigated. However, calculation of the potential impact on risk is more complicated for the latter scenario and will depend on a number of features of the measure, including shape of the population distribution [17]. The new risk factor was independently associated with the outcome; in real-world settings risk factors are often correlated and this will affect their impact on risk estimation. We gave equal weighting to false positive and negative values, which may not always be realistic and may not reflect clinical practice, in which greater weight may be assigned to one type of error.

We examined only a single fracture risk prediction model, although there have been a number of different models proposed [2]; the choice of models will affect AUROC and calibration statistics. Finally, there are other reclassification statistics that have been proposed and may produce different results than the NRI and IDI [23].

## Abbreviations

AUROC: area under the receiver operating characteristic
BMD: bone mineral density
COPD: chronic obstructive pulmonary disease
FRAX^®^: Fracture Risk Assessment Tool
ICD: International Classification of Diseases
MOF: major osteoporotic fracture
NRI: net classification index
IDI: integrated discrimination improvement
RR: relative risk
